# Primary malignant giant cell tumor of the sternum

**DOI:** 10.4322/acr.2021.281

**Published:** 2021-05-25

**Authors:** Mohammed Imaduddin, Pavithra Ayyanar, Mahesh Sultania, Dillip Muduly, Mukund Namdev Sable, Suprava Naik, Sambit Mohanty, Madhabananda Kar

**Affiliations:** 1 All India Institute of Medical Sciences, Department of Surgical Oncology, Sijua, Patrapada, Bhubaneswar, Odisha, India; 2 All India Institute of Medical Sciences, Department of Pathology and Lab Medicine, Sijua, Patrapada, Bhubaneswar, Odisha, India; 3 All India Institute of Medical Sciences, Department of Radiodiagnosis, Sijua, Patrapada, Bhubaneswar, Odisha, India; 4 Prolife Diagnostics, Department of Pathology, Sijua, Patrapada, Bhubaneswar, Odisha, India

**Keywords:** Neoplasms, Giant cell tumors, Sternum

## Abstract

Primary malignant giant cell tumor (PMGCT) is a diagnosis based on the presence of a high-grade sarcomatous component along with a typical benign giant cell tumor (GCT). We report the first case of PMGCT of the sternum in a 28-year-old male with painless swelling over the manubrium sterni. The differential diagnoses of PMGCT and giant cell-rich osteosarcoma were considered. Surgical resection was performed, and the reconstruction was done with a neosternum using polymethyl methacrylate and prolene mesh. At 30 months follow-up, the patient is disease-free.

## INTRODUCTION

Giant cell tumor (GCT) is a benign but locally aggressive neoplasm of mesenchymal origin characterized by the proliferation of osteoclastic multinucleated giant cells in a background of mononuclear cell stroma. Rarely, GCT can undergo a malignant transformation, which may be osteosarcoma, fibrosarcoma, or undifferentiated pleomorphic sarcoma.^1^ This transformation can be either primary or secondary. In primary malignancy of giant cell tumor (PMGCT), synchronous high-grade sarcomatous growth is seen along with GCT. Secondary malignancy in giant cell tumor (SMGCT) is a metachronous sarcomatous growth occurring at the site of previously treated GCT, either by surgery or by radiotherapy.^2^ GCT accounts for 5-6% of all primary bone tumors, with the incidence of primary malignancy constituting 1.6% of all GCT.^1^ ^,^ ^3^ GCT usually occur in the meta-epiphyseal region of long tubular bones, but the axial skeleton and small bones of hand and feet may be rarely involved.^4^ The involvement of sternum by GCT is very rare, with only a few case reports in English literature. We present a case of PMGCT of the sternum, which is the first case reported in English Literature.

## CASE REPORT

**Figure 1:**
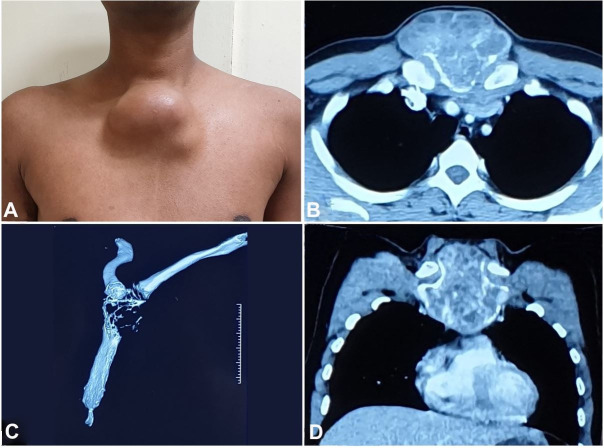
**A** – Clinical photograph of the patient showing a lobulated swelling over the manubrium sterni; **B-D** – Contrast-enhanced computed tomography showing expansile lytic lesion involving manubrium sterni and proximal body of sternum with large heterogeneously enhancing soft tissue component, peripheral rim of calcification and contiguous infiltration of adjacent pectoralis major (B – Axial plane; C – Sternal 3D reconstruction; D **–** Coronal plane).

A 28-year-old male patient presented with a painless, rapidly progressing swelling over the upper sternum for 6 months. On examination, a 9.0×6.0 cm lobulated swelling was noted over the manubrium sterni extending 2 cm above the sternal notch superiorly, sternal angle inferiorly, and just beyond the sternoclavicular joints on both sides laterally. The mass was cystic to hard, and fixed to the underlying bone (Figure 1A). The contrast-enhanced computed tomography (Figure 111D) showed a 9.7×9.0×8.1 cm lobulated expansile lytic lesion involving the manubrium sterni and the proximal body of the sternum with large heterogeneously enhancing soft tissue component, peripheral rim of calcification, and contiguous infiltration of adjacent pectoralis major muscle.

The histological examination of the biopsy showed predominantly osteoclastic giant cells, pleomorphic stromal cells, scattered bizarre cells, and many atypical mitotic figures. Also, it showed an acellular eosinophilic material in a filigree pattern resembling osteoid. Based on these features, the differential diagnosis of high-grade giant cell-rich osteosarcoma (GCRO) and PMGCT was considered.

**Figure 2:**
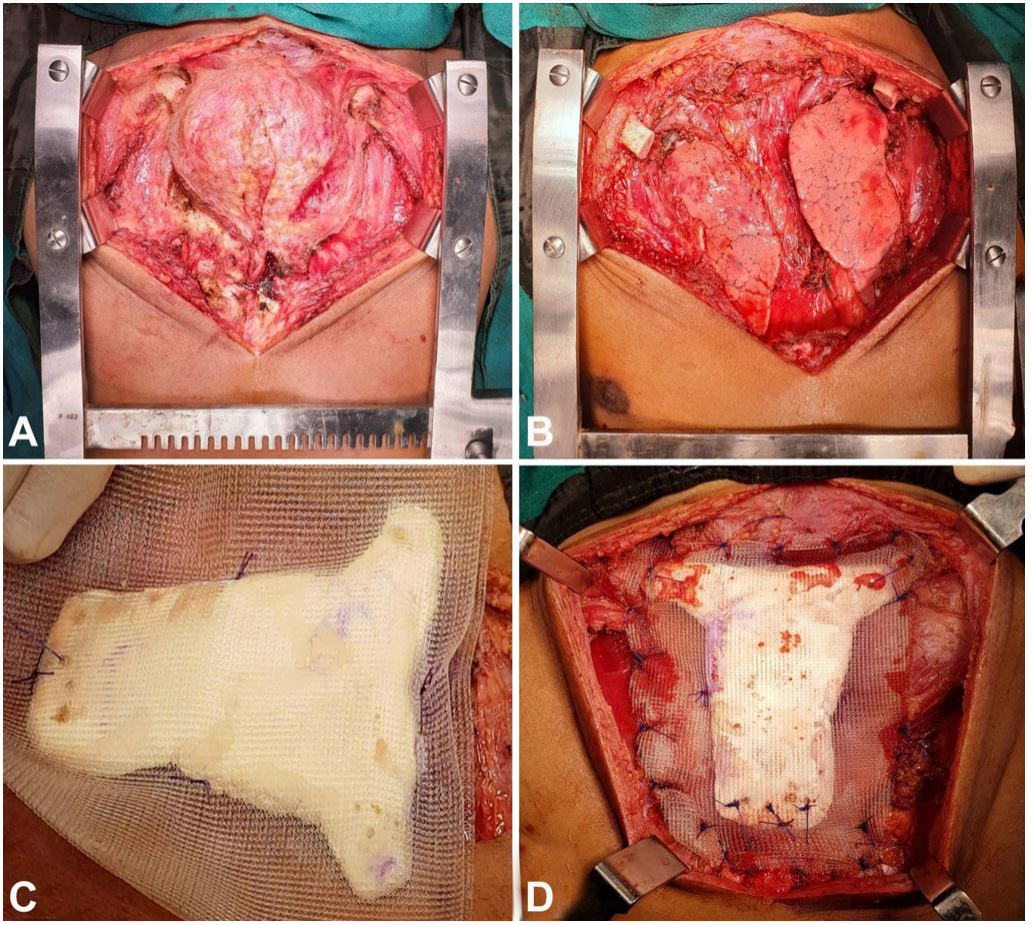
**A –** Intra-operative image showing the tumor extent; **B –** Chest wall defect post-resection; **C –** Sternal prosthesis prepared using PMMA; **D –** Reconstruction using sternal prosthesis.

The resection of the manubrium sterni mass was done by including the first three ribs bilaterally 3cm lateral to the costochondral junction, sternum at the level of 4th rib inferiorly, and clavicles 3cm lateral to the sternoclavicular joints. The left pectoralis major myofascial (PMMF) advancement flap was mobilized and placed over the defect to cover the contents of the anterior mediastinum. A T-shaped sternal prosthesis was prepared using polymethyl methacrylate (PMMA) shaped to fit in the bony defect. Holes were drilled in the prosthesis at the lower and upper ends, and corresponding holes were drilled in the upper end of the sternal remnant and the medial cut ends of clavicles. The prosthesis was then placed between two layers of prolene mesh, and the sternal prosthesis assembly was sutured in place by using prolene sutures and passing it through the corresponding drilled holes. Right PMMF flap was mobilized over the sternal prosthesis assembly and skin sutured after placing a closed subcutaneous drain. Post-operatively, the patient recovered well without any major complications (Figure 2AD).

**Figure 3:**
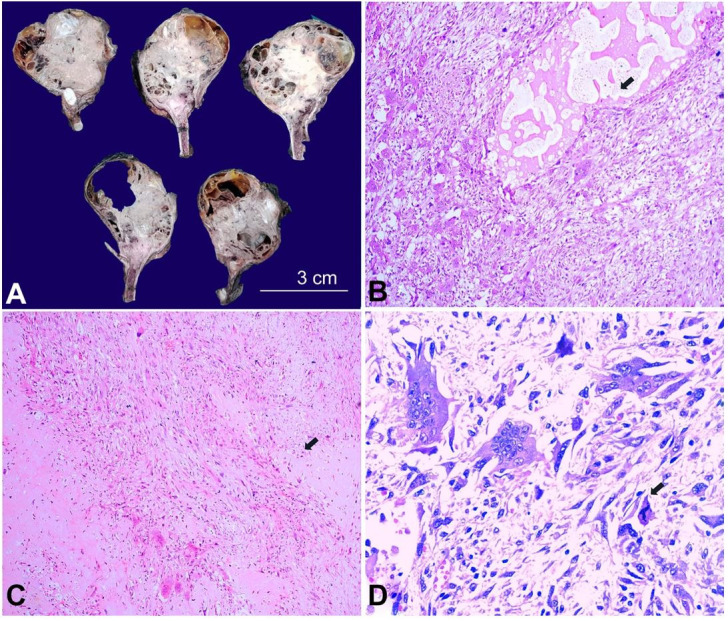
**A –** Photograph of wide local excision of the sternal mass showing a solid cystic tumor of tan-white to tan-grey solid mass with areas of hemorrhage; (**B-D**) – Histopathological examination showed a cellular, osteoclastic cell-rich lesion with aneurysmal bone cyst-like areas (B, H& E, 100X), extracellular osteoid formation (C, H& E, 100x) and marked cellular and nuclear pleomorphism with atypical mitotic figures (D, H& E, 400x).

Grossly, outer surface of the surgical specimen was tan-white to tan-grey with an intact bosselated surface. Cut surfaces revealed a 10.0×10.0×9.5 cm tumor, with solid and tan-white cystic surfaces; and cystic components containing thick gelatinous material. Focal areas of hemorrhage, congestion, and calcification were noted (Figure 3A).

Microscopically, the tumor had increased cellularity, with spindling of mononuclear cells. The mononuclear cells exhibited marked nuclear pleomorphism and presence of atypical mitotic figures (8 mitoses per 10 high power fields). Foamy histiocytic infiltration, large secondary aneurysmal bone cyst-like areas, and areas of necrosis were noted. Focal areas showed cartilage and osteoid within the spindle cell component (Figure 33D). The tumor infiltrated beyond the cortex and extended into the surrounding soft tissue. All resection margins were free of tumor. Based on the histomorphology, a final diagnosis of PMGCT was rendered with osteosarcoma-like areas.

The case was discussed in the institutional tumor board and planned for follow-up without adjuvant therapy. After 30 months of follow-up, the patient is alive and disease-free.

## DISCUSSION

The present case represents a PMGCT at an unusual location, the sternum. The differential diagnosis of giant cell-rich osteosarcoma created a diagnostic dilemma that was resolved by radiological correlation. The unusual location and extent of the lesion posed a surgical challenge for reconstruction. Due to the paucity of data. The role of an adjuvant therapy was debatable.

Giant cell tumors of bone are relatively uncommon and are mostly located around the knee (distal femur or proximal tibia).^3^ It may, however, rarely present in the sternum. To date, 12 cases of sternal giant cell tumors have been reported, none of which were malignant (Table 1: Review of Sternal Giant cell tumor cases reported in literature).

**Table 1:** Review of Sternal Giant cell tumor cases reported in literature

author	Age (y)/Gender	Symptom	Location	Size (cm)	Nature	Surgery	Reconstruction	Follow-up (m)
Sundaram et al.^13^	55/ M	Painless swelling	Manubrium	-	B	STE	None	-
Bay et al.^14^	49/ F	Pain	Manubrium	3.9×3.2	B	STE	Prosthesis	60
Segawa et al.^15^	55/ M	Pain	S body	3.5×3.0	B	Ctg	PMMA filling, prolene mesh and titanium mesh plate	12
Imai et al.^16^	45/ M	Pain	S body	8.4×4.5×2.5	B	STE	PMMA Prosthesis	12
Futani et al.^17^	53/ F	Pain	S body	8.0×4.0×2.5	B	Ctg	PMMA filling	84
Abate et al.^18^	28/ M	Painful swelling	S body	6.4×4.3×4.4	B	STE	PMMA Prosthesis, prolene mesh, bilateral PMMF	5
Faria et al.^19^	74/ F	Painful swelling	S body	12×7.5×4.5	B	STE	Fascia lata	5
Engel et al.^10^	32/ M	Painful swelling	Manubrium	7.6×5.1×4.7	B	STE	Gore Dualmesh Plus	10
Traibi et al.^20^	34/ F	Painful swelling	S body	14.0×9.0×8.0	B	STE	PMMA Prosthesis, prolene mesh, Bilateral PMMF	-
Wang et al.^21^	53/ F	Painful swelling	S body	3.0×1.6×1.5	B	STE	PMMA Prosthesis, bilateral PMMF	12
Muramatsu et al.^22^	16/ M	Pain	S body	5.0×3.0	B	Ctg	beta-tricalcium phosphate filling	12
	44/ M	Painless mass	S body	8.0×3.0	B	Ctg	Posterior cortical wall preserved	24
Index case	28/M	Painless mass	Manubrium	14×13.5×9	Mal	Subtotal sternectomy	PMA prosthesis, prolene mesh, bilateral PMMF	30

M = Male; F = Female; B = benign; Ctg = curettage; S body = sternal boby; Mal = malignant; m = months; PMMA = polymethyl methacrylate; PMMF = pectoralis major myofascial; STE = subtotal sternectomy; y = years.

Giant cell tumors of bone are relatively uncommon and usually located around the knee (distal femur or proximal tibia).^3^ It may, however, rarely present in the sternum. We did a thorough search of the Pubmed Database using the MESH terms sternum, neoplasms and giant cell tumors. Twenty-two results were found, which were reviewed. Among these, 12 cases of sternal giant cell tumors were found. However, all of these reported cases were benign, making the present report the first case of primary malignant giant cell tumor of the sternum in English literature (Table 1).

Malignancy in giant cell tumor of bone was first described by Stewart in 1938.^5^ Over the years, “Malignant giant cell tumor” has been used as a non-specific term to describe GCT with a variable degree of anaplasia, metastasizing benign GCT, malignant fibrous histiocytoma with multinucleated cells and locally aggressive benign GCT. Jaffe^6^ and Dahlin et al.^2^ promulgated the concept of malignant giant cell tumors wherein there should be histological evidence of a giant cell tumor along with the malignant sarcomatous stromal component. Unni et al.^3^ proposed the term “malignancy in giant cell tumor” and classified it into primary and secondary. Primary malignancy in GCT has a sarcoma component de novo juxtaposed on a giant cell tumor whereas secondary malignancy in GCT is a sarcoma at the site of previously treated (surgery or radiotherapy) histological proven GCT.

The diagnosis of primary malignancy in GCT is challenging. The usual features of malignancy like cellular pleomorphism, nuclear atypia, increase in mitotic activity, necrosis, and vascular emboli are not uncommon in giant cell tumors and do not satisfy the criteria to diagnose primary malignancy in GCT.^6^ The presence of a high-grade sarcomatous component is an essential must for the diagnosis.^7^ The presence of areas with features of benign GCT within the tumor can misguide the diagnosis if the malignant portion is missed on the initial biopsy.

Giant cell-rich osteosarcoma is an important differential diagnosis that can be difficult to differentiate. A diaphyseal or meta-diaphyseal location, osteoclast-like giant multinucleated cells, direct osteoid formation by malignant giant cells, and absence of typical features of benign GCT aid in diagnosis.^8^ It is pertinent to differentiate PMGCT from giant cell-rich osteosarcoma as the need for neoadjuvant chemotherapy differs between the two – recommended in giant cell-rich osteosarcoma whereas PMGCT is treated by surgical resection without neoadjuvant chemotherapy.

The initial biopsy in our case showed osteoclastic giant cells, pleomorphic stromal cells, and typical mitotic figures with few foci of osteoid-like material. Although the features were suggestive of primary malignancy in GCT, the presence of osteoid made it difficult to differentiate with giant cell-rich osteosarcoma. The sternal location of the tumor, which is rare for both diagnoses, could not aid in the diagnosis. Bathurst et al.^9^ described the radiological pattern of giant cell-rich osteosarcoma as a predominantly lytic lesion with ill-defined margins, weak periosteal reaction, and absence of soft tissue component.^9^ The radiological findings of lack of osteoid and periosteal reaction with the presence of soft tissue component in our case were correlated with the biopsy findings, and the diagnosis of PMGCT was favored.

Due to the rarity of the diagnosis and lack of proper consensus on a definition, the choice of treatment is not well defined in PMGCT. Surgical resection is usually indicated but the role of chemotherapy is controversial. The sternal location of the tumor in our case was a surgical challenge. Of the 12 cases of sternal GCT reported, the body of the sternum was involved in 9 cases (75%) and 3 out of 12 cases (25%) involved the manubrium. Subtotal sternectomy was done in 8 cases (66.6%). The 3 patients with manubrium involvement underwent subtotal sternectomy. The malignant nature of the tumor and the need to sacrifice bilateral sternoclavicular joints posed a challenge to surgery and reconstruction. Engel et al.^10^ performed a subtotal sternectomy with the division of bilateral sternoclavicular joints and disarticulation of the body-manubrium joint. Rigid reconstruction was done using mesh, and soft tissue coverage was done using bilateral pectoral advancement flaps. We preferred to create a neosternum using PMMA for rigid construction as the defect size was large and only mesh might not have given adequate support.

The role of adjuvant therapy in PMGCT is not well defined. A retrospective study reported a better one-year survival with surgery and adjuvant chemotherapy compared to surgery alone.^11^ However, this advantage was not projected in five-year survival rates and actuarial survival curves. Radiotherapy has been reported to be associated with better prognosis and is suggested as adjuvant therapy to surgery.^12^ Adjuvant radiotherapy was considered in our case but the potential risks of toxicity to the heart and lungs in the radiation field outweighed the benefit of therapy.

## CONCLUSION

Primary malignancy in giant cell tumor of the sternum is a rare diagnosis. The presence of a sarcomatous component juxtaposed on typical giant cell tumor helps in diagnosis. Surgical resection with negative margins should be the goal of treatment. Fear of the size of the defect should not be a hindrance to resection. Reconstruction can be done using a prosthesis or a mesh depending on the magnitude of the defect and the stability of the chest wall. Adjuvant chemo or radiotherapy can be decided on an individual basis based on the principles of precision medicine.
